# Consideration of substance use in compensation and pension examinations of veterans filing PTSD claims

**DOI:** 10.1371/journal.pone.0210938

**Published:** 2019-02-06

**Authors:** Rebecca L. Jankowski, Anne C. Black, Christina M. Lazar, Bradley R. Brummett, Marc I. Rosen

**Affiliations:** 1 VA Central Western Massachusetts Healthcare System, Leeds, Massachusetts, United States of America; 2 VA Connecticut Healthcare System, West Haven, Connecticut, United States of America; 3 Yale University, New Haven, Connecticut, United States of America; Hunter Holmes McGuire VA Medical Center, UNITED STATES

## Abstract

Veterans filing claims that service-induced PTSD impairs them worry that claims examiners may attribute their difficulties to conditions other than PTSD, such as substance use. Substance use commonly co-occurs with PTSD and complicates establishing a PTSD diagnosis because symptoms may be explained by PTSD alone, PTSD-induced substance use, or by a substance use condition independent of PTSD. These alternative explanations of symptoms lead to different conclusions about whether a PTSD diagnosis can be made. How substance use impacts an examiner’s diagnosis of PTSD in a Veteran’s service-connection claim has not been previously studied. In this study, we tested the hypothesis that mention of risky substance use in the Compensation & Pension (C&P) examination would result in a lower likelihood of service-connection award, presumably because substance use reflected an alternative explanation for symptoms. Data were analyzed from 208 Veterans’ C&P examinations, medical records, and confidentially-collected research assessments. In this sample, 165/208 (79%) Veterans’ claims were approved for a mental health condition; 70/83 (84%) with risky substance use mentioned and 95/125 (76%) without risky use mentioned (p = .02). Contrary to the a priori hypothesis, Veterans with risky substance use were *more* likely to get a service-connection award, even after controlling for baseline PTSD severity and other potential confounds. They had almost twice the odds of receiving any mental health award and 2.4 times greater odds of receiving an award for PTSD specifically. These data contradict assertions of bias against Veterans with risky substance use when their claims are reviewed. The data are more consistent with substance use often being judged as a symptom of PTSD. The more liberal granting of awards is consistent with literature concerning comorbid PTSD and substance use, and with claims procedures that make it more likely that substance use will be attributed to trauma exposure than to other causes.

## Introduction

In Fiscal Year (FY) 2016, the Veterans Benefits Administration (VBA) branch of the Department of Veterans Affairs (VA) approved nearly 100,000 new claims for mental health conditions caused or worsened by military service. Over half of these (53,983) were new claims for Posttraumatic Stress Disorder (PTSD). As of FY 16, there were a total of 887,899 Veterans compensated for service-connected PTSD, the most commonly compensated mental health condition and the third most compensated condition overall (behind only tinnitus and hearing loss).[1] Having a documented service-connected disability has several important benefits, one of which is eligibility to receive monthly disability compensation, which varies by the degree of disability (rated between 0–100%). The modal degree of compensation for service-connected mental health conditions (PTSD and other mental disorders) in FY 16 was 70%, whereas the other 15 most prevalent compensated disorders were each most often compensated at or below 20%.[1]

Service-connection claims are adjudicated by the VBA. Determinations are largely based on the results of an examiner’s report. Examiners for mental health claims such as PTSD are typically licensed psychologists or board-certified psychiatrists.[2, 3] Starting in 2011, examiners submitted their reports using a standardized, detailed form called the Disability Benefits Questionnaire (DBQ).[3] The DBQ usually contains information elicited during an interview and examination of the Veteran and from a detailed records review. The available documents comprise the VA Claim-File (C-file), which may include military personnel records, statements from the Veterans and others, service treatment records, and non-military treatment records. The examiner’s report describes the history of the claimed condition, reported symptoms, the extent of social and functional impairments, examiner observations, and diagnosed psychiatric disorders. If more than one mental health diagnosis is present, examiners are asked to describe the degree of impairment attributable to each disorder. At the end of the report, the examiner offers a judgment as to whether the disorder was “at least as likely as not (50% or greater probability)” caused or worsened by the Veteran’s military service. VBA adjudicators then apply a rubric to the impairment described by the examiner to rate the degree of disability.

High proportions of individuals with PTSD also have substance use disorders (SUD).[4, 5] Among Veterans with PTSD receiving care at VA, approximately 27% also have a SUD and, according to the National Center for PTSD, almost 1 in 3 Veterans seeking addiction treatment have comorbid PTSD.[6] In a large nation-wide study of OEF/OIF first-time VA users between 2001 and 2010, Veterans with a diagnosis of PTSD and/or depression were 3–4.5 times more likely to also meet criteria for alcohol and/or drug use disorders compared to Veterans without those diagnoses.[7]

In Veterans with significant substance use histories, determinations of PTSD related to military service are complicated by the possibility that an independent substance use condition accounts for symptoms and PTSD does not. Withdrawal symptoms from alcohol may include anxiety, irritability, sleep disturbance, and/or exaggerated startle response while withdrawal from a stimulant, such as cocaine, may include hypervigilance, paranoia, anxiety, and sleep and mood disorders.[8–10] These substance-related symptoms overlap with DSM-5 Criterion E for a PTSD diagnosis—arousal symptoms, such as irritability, hypervigilance, heightened startle response, and sleep disturbance.[10] Individuals with comorbid PTSD and SUD often have more severe PTSD symptoms[11] than those with either disorder alone, and PTSD and SUD symptoms appear to co-vary.[12, 13] It is also problematic to determine the extent to which social and occupational impairments are due to PTSD symptoms and/or substance use, and such impairment is central to the rubric by which the VBA rates service-connection claims.[14]

Several explanations have been offered for the co-occurrence of PTSD and substance use, depending on the sequence in which they occur. The occurrence of substance use before PTSD has been posited to relate to people with substance use problems being in riskier situations, thus increasing the risk of exposure to trauma.[15, 16] Another explanation for PTSD occurring in people with substance use disorders is that vulnerability to the development of PTSD may increase with prolonged substance use. In other Veterans, PTSD symptoms precede substance use. This sequence has been explained by individuals with PTSD using substances to relieve trauma symptoms.[17–20] Another explanation is a shared vulnerability, genetic or otherwise, to developing both PTSD and substance use disorder.[21–24]

Disentangling a PTSD diagnosis from a substance use diagnosis can be challenging and is further complicated by a 1990 law precluding compensation for disabilities resulting from a Veteran’s willful abuse of alcohol or drugs (38 CFR § 1110). The interpretation of the statute has been contested over time. In a 1998 case, *Barela v*. *West*, the Board of Veterans’ Appeals held that “service connection for alcohol and drug abuse, claimed as secondary to service-connected PTSD with depression, is prohibited by law.”[25] However, the US Court of Appeals for Veterans Claims reversed and remanded the Board’s decision, holding that the Board went too far in their interpretation of this statute and that § 1110 prohibits compensation for disabilities related to substance abuse but it does not bar an award of service-connection. Then, in 2001, the US Court of Appeals for the Federal Circuit, in *Allen v*. *Principi*, rejected the *Barela* court’s interpretation, calling it erroneous. The Court of Appeals held that the statute did not preclude a Veteran from receiving compensation for a substance abuse disability that arose from a service-connected disability or from using substance use disabilities as evidence of the increased severity of a service-connected disability. However, Veterans could only be compensated for a substance abuse disorder if they could establish that their substance abuse was caused by their primary service-connected disorder and that it was not “willful” abuse.[26] Given the high co-morbidity of PTSD and substance use, along with the contentiousness of substance use in service-connection decisions, it is understandable that service-connected Veterans have expressed worry that they must be careful what they present in the claims process [27] and during VA treatment.[28]

In this paper, we examined the association between mentions of risky substance use in examiners’ DBQ reports and service-connection determinations in a carefully-characterized cohort of Veterans applying for initial service-connection for PTSD. Mentions of risky substance use in examiners’ reports reflect both the substance use information examiners had access to and their choices about how to incorporate those data into their report. Because substance use provides an alternative explanation for some symptoms of PTSD, we hypothesized that mentions of risky substance use in the Compensation & Pension (C&P) examination would be associated with lower PTSD claim approval rates. We posited that this association would remain after controlling for characteristics differentiating people with and without risky substance use and variables associated with receipt of service connection.

## Methods

### Human subjects

Data were analyzed from a subset of 208 participants (those enrolled in Connecticut between April 2013 and July 2016) from a multi-site, randomized clinical trial focused on fostering treatment engagement among Veterans applying for service-connection for PTSD (NCT01597856). Participants were post-9/11 era Veterans who were scheduled for an initial PTSD exam in support of their C&P claim. Veterans in the parent study (the clinical trial) completed the assessments used in this analysis before they had received any study-related intervention. The analyses also included data extracted from participants’ electronic health records and from their C&P examinations. All participants provided written informed consent prior to undergoing any research procedures. This research was approved by the VA Central Institutional Review Board.

### Data from face-to-face assessments from the clinical trial

Participants completed a battery of assessments with a research assistant upon study enrollment. An initial questionnaire, designed to characterize study participants, asked about basic demographics, military history, psychiatric history, and recent employment history. The questionnaire included a four-question screen for traumatic brain injury (TBI) used by the VA.[29]

Exposure to combat was measured by a single, dichotomous item asking whether the Veterans was “at any time in a combat zone or in life-threatening situations while in military service”.

PTSD was assessed using the Clinician-Administered PTSD Scale for DSM-IV (CAPS-IV).[30] Before the development of CAPS for DSM-5, CAPS-IV was a widely-used observer-rated clinical interview used to derive a continuous index of PTSD symptom severity and diagnosis. Respondents identify up to three traumatic events to keep in mind during the interview and then answer questions about the frequency and intensity of 17 PTSD symptoms listed in the DSM-IV that they have experienced in the past month. Following assessment scoring rules, the sum of symptom frequency (scaled 0, never– 4, daily or almost every day) and intensity (scaled 0, none– 4, extreme) for all 17 items (scaled 0–136) was calculated. PTSD diagnoses were then determined for Veterans who met two rules: (1) the “F1/I2” rule requiring frequency of at least 1 and intensity of at least 2 for any given symptom to be considered present, and then requiring the presence of at least one reexperiencing symptom, three avoidance and numbing symptoms, and two hyperarousal symptoms; and (2) a total severity score (i.e., sum of all frequency and intensity ratings for all items) ≥45.[31] This combination of rules was used in a previous large multisite study of women Veterans.[32]

The CAPS-IV was administered by research assistants with previous experience working on studies of Veterans with mental health issues. Research assistant training included observation of videotaped CAPS interviews obtained from the National Center for PTSD,[33] with additional face-to-face training by a senior CAPS expert at VA. Rating issues arising during CAPS-IV administration were discussed during weekly study group meetings with study investigators.

Depression was assessed using the Beck Depression Inventory—II (BDI-II).[34] This self-reported assessment consists of 21 statements reflecting DSM-IV somatic symptoms and cognitive factors associated with depression. Respondents rank each statement on a 0–3 severity scale, and total sum score is calculated. The instrument has shown excellent receiver-operating characteristics in identifying depressed Veterans from a cohort of Veterans with TBI and frequent comorbid PTSD.[35]

Substance use was assessed by self-report using a timeline follow-back (TLFB) substance use calendar.[36] Participants were presented with a calendar and used key dates to recall their use of alcohol, illegal drugs, and prescribed opiates each day in the prior twelve weeks. Participants were classified as having risky substance use if they reported (1) >4 drinks/day or >14 drinks/week for men or >3 drinks/day or >7 drinks/week for women, (2) use of an illicit drug, or (3) misuse of a prescribed opiate. Our classification of these levels of alcohol use as risky is based on the National Institute on Alcohol Abuse and Alcoholism (NIAAA) definition.[37]

### Data extracted from electronic health record and compensation and pension examination

Data from each Veteran’s electronic health record and PTSD C&P examination were extracted by a single reviewer (RJ). All clinical notes within a year before each Veteran’s C&P exam and the examiner’s written report were reviewed for any documentation of alcohol and/or drug use. Chart notes and examiner reports indicating a discussion about substances that were used, type of substances, amount and frequency of use, any substance use diagnoses, and any consequences of substance use were transcribed.

Extracted data were then reviewed by two raters (CL and RJ) blind to service-connection outcomes and a determination was made as to whether the notes indicated risky substance use or not using the criteria above. Because chart and exam documentation of substance use was ad hoc and unstructured, we defined rules for scoring mentions of substance use as risky or not. Risky alcohol use was rated if the chart or exam contained (a) a diagnosis or rule-out diagnosis of alcohol abuse/dependence made in the note, or (b) a note indicated a “risky” level of drinking (e.g. descriptive documentation of “excessive alcohol use” or amounts that were at or above NIAAA-defined risky levels). A determination of no risky alcohol use was made if there was a note indicating no alcohol was used, or if a note indicated alcohol was used but at a level below what is considered risky. A determination of risky drug use was made if there was a note indicating an illicit drug was used within the past six months or a diagnosis of a drug use disorder (e.g. drug abuse or dependence) was documented, not including Nicotine Use Disorder. A determination of no risky drug use was also made if there was a note in the chart or exam that indicated no drugs were used or if a note indicated a specific substance was used but had no additional information about it that would indicate current use (such as frequency and amount used). In cases where risky alcohol and/or drug use was noted as “in remission,” Veterans were rated as “not risky” if remission was noted as “sustained remission” for a year or more and rated as “risky” if remission was noted as “early.”

### Data extracted from Veterans Benefits Administration records

Data from VBA rating determination letters were extracted, and included the service-connected conditions for which the Veteran was awarded compensation and the degree (%) of award.

### Data analysis

Data analysis proceeded in several stages. First, Veterans with and without risky substance use mentioned in their C&P examinations were compared on demographic and personal characteristics, military service, and mental health diagnoses to identify group differences that might confound model results because of their association with the exam outcomes. The characteristics compared between groups are listed in Table 1. Although groups were also compared on self-reported alcohol and substance use, these variables were not considered for inclusion in models of exam outcomes because this information was not available to examiners. The group comparison on alcohol and substance use serves as validation of the primary predictor, risky substance use mentions.

**Table 1:** Characteristics of Veterans with risky and non-risky substance use documented in C&P exam.

	Not Risky n = 125	Risky n = 83	DF	Statistic	*p* value
Age (median, IQR)	31 (27–39)	30 (26–35)		MWU = 4620	0.18
Sex (male)	102 (82%)	77 (93%)	1	X^2^ = 5.2	0.02
Race/Ethnicity			3	X^2^ = 2.4	0.50
White	82 (66%)	57 (69%)			
Black	16 (13%)	14 (17%)			
Hispanic	20 (16%)	10 (12%)			
Other	7 (6%)	2 (2%)			
Marital status			2	X^2^ = 3.9	0.14
Married	36 (29%)	26 (31%)			
Single (past married)	49 (39%)	22 (27%)			
Single (never married)	40 (32%)	35 (42%)			
Served in a Combat Zone	107 (86%)	78 (94%)	1	X^2^ = 3.558	0.06
Education (median, IQR)	14 (13–16)	14 (13–15)		MWU = 4758	0.30
Employed Full-time	64 (51%)	46 (55%)	1	X^2^ = 0.4	0.55
Years Active Duty (median, IQR)	5.0 (3.5–9.7)	4.0 (3.0–7.0)		MWU = 4316	0.04
Heath Insurance	n = 123	n = 79	2	X^2^ = 0.3	0.88
Private	65 (53%)	39 (49%)			
VA w/o private	50 (41%)	34 (43%)			
Public w/o other	8 (7%)	6 (8%)			
Positive TBI Screen	n = 124 73 (59%)	n = 83 41 (49%)	1	X^2^ = 1.8	0.18
Depression (BDI-II mean ± SD)	n = 123 22.8 ± 11.8	n = 83 24.6 ± 10.5	204	t = -1.147	0.25
Depression Classification	n = 123	n = 83	2	X^2^ = 1.9	0.39
Low (0–16)	40 (33%)	20 (24%)			
Moderate (17–30)	47 (38%)	38 (46%)			
Significant (>30)	36 (29%)	25 (30%)			
PTSD diagnosis (CAPS-IV criteria with ½ rule met and severity ≥ 45)	n = 124 85 (69%)	n = 83 60 (72%)	1	X^2^ = 0.3	0.57
PTSD severity (mean ± SD)	60.4 ± 20.9	58.4 ± 22.2	206	t = .661	0.51
Substance use (from TLFB)					
Any risky alcohol use	64 (41%)	60 (72%)	1	X^2^ = 9.2	<0.01
Any risky drug use	24 (19%)	29 (35%)	1	X^2^ = 6.5	0.01
Any risky substance use	73 (58%)	67 (81%)	1	X^2^ = 11.3	<0.01

Next, using logistic regression modeling, we tested the unconditional relationship between risky substance use mentioned in the C&P exam and two C&P exam outcomes. A binomial logistic regression model was used to estimate the relationship between risky substance use mention and the dichotomous outcome, C&P award decision (awarded compensation or not). A multinomial logistic regression model estimated the relationship between risky substance use mention and the polytomous outcome, type of C&P award (compensation for service-connected PTSD, compensation for other mental health condition, or no award for any mental health condition).

Finally, the *conditional* relationship between risky substance use mentioned in the C&P exam and each C&P exam outcome (award decision and type of award) was tested in separate logistic regression models, controlling for covariates. Covariates included in each model were those that differed significantly between the risky and no-risky substance use groups. Additional covariates were included in the models regardless of their statistical significance in between-group comparisons because of their association in previous studies with C&P award determinations; these variables were African-American identity[38–40] and full-time employment.[41] Models also controlled for PTSD severity as measured by the CAPS given the central role of PTSD severity in PTSD C&P award decisions[42] and the well-documented association between PTSD and substance use.[4–7] Including PTSD severity as a covariate allowed us to understand if examiners considered substance use after controlling for differences in PTSD symptoms. Because the timing of the CAPS assessment with respect to the C&P exam differed across participants, the number of days elapsed between C&P exam and CAPS assessment was tested as a covariate but was not included in the final model because it was not a statistically significant predictor.

In all regression models, standard errors were corrected for the effect of clustering of Veterans within C&P examiners on claim outcome (ICC = .16). Regression models were conducted using Mplus version 8 software.[43] All statistical tests were evaluated at a significance level of .05.

## Results

### Study participants

A total of 224 Veterans were enrolled in the parent study from Connecticut. Of those, 208 (93%) had complete data on all predicting variables included in the final model. Veterans were mostly male (86%) and white (67%). Comparable proportions were married (30%), divorced (34%), and never married (36%). Over two thirds (70%) of the sample had a PTSD diagnosis derived from the research assistant-administered CAPS, with a mean (± standard deviation) severity score of 59.6 ± 21.4, and a mean BDI-II score of 23.5 ±11.3. Over two thirds of Veterans (140/208) reported risky substance use on the TLFB calendar within the past twelve weeks. Among those with risky use, 87/140 (62%) had only risky alcohol use, 16/140 (11%) had only risky drug use, and 37/140 (26%) had both risky alcohol and drug use. On average, participants completed the CAPS-IV during research assessments conducted 15.3 days after their C&P examination (median = 13.0 days; SD = 15.7). The majority (79%) of Veterans were awarded compensation for this filed claim, 57% for PTSD and 23% for another mental health condition. Of the 165 Veterans awarded, 32% were granted 30% service connection, 35% were granted 50% service connection, and 21% were granted 70% service connection.

A total of 83 of the 208 Veterans (40%) had risky substance use noted in their C&P exam (n = 60 had only risky alcohol use, n = 14 had only risky drug use, and n = 9 had both risky alcohol and drug use).

Compared to Veterans with no risky use mentioned in their exams, Veterans with risky substance use were more likely to be male (93% vs.82%, x^2^ = 5.2, p = .02) and reported significantly fewer years of active duty (median 4.0 years vs. 5.0 years, MWU = 4316, p = .04). The groups differed significantly in the expected direction on any risky alcohol use, any risky drug use, and any risky alcohol or drug use. The groups did not differ significantly by age, race/ethnicity, marital status, education, service in a combat zone, full-time employment, health insurance, TBI history, BDI-II depression scores, PTSD symptom severity or PTSD diagnosis, as described in Table 1.

### Binomial and multinomial logistic regression analyses

The models of service-connection for any mental health condition are presented in Table 2. The model with covariates includes variables that significantly differed between Veterans with and without risky substance use in their claims reports (sex and years of active duty), characteristics whose association with C&P award was noted in previous studies (African-American identity[38–40], full-time employment status[41]), and PTSD severity as measured by the CAPS-IV. As expected, PTSD severity was a significant predictor of being awarded service-connection. For every 1-point increase in PTSD severity, the odds of service connection increased by 3% (p = .01). In this model controlling for other covariates, having risky substance use documented by the C&P examiner was significantly positively associated with being awarded service-connection. Compared to Veterans without documentation of risky substance use, Veterans with risky substance use documented had almost two times greater odds of being service-connected (OR = 1.99, p = .02).

**Table 2:** Binomial logistic regression models of any compensation award.

	Model without Covariates			Model with Covariates		
Predictor	Estimate	OR	95% CI	Estimate	OR	95% CI
Risky Substance Use	0.53	1.70	1.08, 2.67*	0.69	1.99	1.14,3.49*
Male				-0.45	0.64	0.28,1.45
PTSD severity				0.03	1.03	1.01,1.05*
Full Time Employment				0.31	1.36	0.60,3.09
African American				-0.06	0.94	0.37,2.40
Years Active Duty				0.02	1.02	0.94,1.11

* = *p*<0.05

The next analysis considered more specifically what type of service-connection award was associated with mentions of risky substance use. Table 3 shows the results of the multinomial logistic regression model estimating the effects of predictors on compensation for service-connected PTSD and service-connection for other mental health condition relative to no award for any mental health condition. As expected, PTSD severity was positively associated with being awarded service-connection for PTSD (OR = 1.04, p < .01). Mention of risky substance use was also associated with greater odds of an award for PTSD (OR = 2.43, p < .01). Neither PTSD severity nor mentions of risky substance use was associated with being awarded service-connection for another mental health condition.

**Table 3:** Multinomial logistic regression of compensation award for PTSD or any mental health condition vs. no award.

	PTSD award vs. no award						Other MH award vs. no award					
	Model without Covariates			Model with Covariates			Model without Covariates			Model with Covariates		
Predictor	Estimate	OR	95% CI	Est	OR	95% CI	Estimate	OR	95% CI	Est	OR	95% CI
Risky Substance Use	0.67	1.95	1.23, 3.09**	0.89	2.43	1.34, 4.42**	0.18	1.19	0.53, 2.66	0.33	1.38	0.60, 3.20
Male				-0.19	0.82	0.32, 2.13				-0.97	0.38	0.13, 1.08
PTSD severity				0.04	1.04	1.02, 1.07**				0.00	1.00	0.97, 1.02
Full Time Employment				0.50	1.65	0.70, 3.89				-0.04	0.96	0.33, 2.84
African American				-0.01	0.99	0.37, 2.66				-0.13	0.88	0.25, 3.04
Years Active Duty				0.02	1.02	0.95, 1.10				0.02	1.02	0.91, 1.14

** = *p*<0.01

### Discussion

To our knowledge, this is the first study to examine the impact of Veteran substance use on service-connection claim outcome. The main finding was that documentation of risky substance use in examiner reports was significantly associated with receiving a service-connection award for PTSD. The impact of mentioned risky substance use remained significant, even after controlling for PTSD severity, sex, being African-American, and full-time employment status.

A possible clinical explanation for the effect of risky substance use mentions is that substance use is interpreted in the claims process to be a symptom of PTSD. Rates of PTSD, depression, and substance use are significantly higher following deployment to a combat zone compared to pre-deployment,[44, 45] and Veterans with PTSD experience more severe drug and alcohol abuse problems compared to Veterans without PTSD.[46] Much of the literature suggests that substance use typically parallels the onset and severity of PTSD symptoms[12, 47] and that PTSD symptoms more often precede the development of substance use disorders[5, 17, 48–50] than vice versa, a finding that has been speculatively suggested to reflect substance use being a self-medication for PTSD symptoms.[11, 12, 17, 20, 51] Furthermore, some evidence suggests that substance use may be related to specific PTSD symptom clusters.[11, 52]

Another possible explanation of risky substance use mentions impacting PTSD service-connection is that VBA’s procedures contribute to the attribution of symptoms to PTSD rather than substance abuse. Fundamentally, VBA raters are instructed to look for: (1) credible evidence of a stressor; (2) a medically-derived diagnosis of PTSD; and (3) a link between the stressor and the claimant’s symptoms. The fact that VBA procedures emphasize trauma and not substance use might lead examiners to connect symptoms to traumatic experiences first. The search for alternative explanations of post-stressor difficulties, such as substance use, receives less emphasis. Additionally, the DBQ prompts examiners to investigate Veterans’ substance use “pre-military, military, and post-military.” This prompt suggests that the timing of the substance use—before, during, or after service—is relevant. Furthermore, examiners are instructed to give Veterans the benefit of the doubt in resolving uncertain claims[3, 53, 54], and it is possible that examiners are emphasizing the role of trauma when trying to resolve the nebulous question of what impairment is caused by PTSD and what is caused by substance use.

In the multinomial regression model, documentation of risky substance use was associated with PTSD awards, but not with other mental health awards, compared to no award. The category “other mental health disorders” encompassed a variety of conditions such as Adjustment Disorder, Depression, and Anxiety, with each represented by a small number of Veterans, thus limiting the power to make inferences about any specific condition.

We have shown in a prior study of Veterans filing service-connection claims that fiscal claims concerns are especially important to Veterans with substance use.[55] High proportions of surveyed Veterans and Veterans Service Officers have endorsed the belief that Veterans have to be guarded about what information they disclose during their Compensation exams.[56] The claims process has been described as a barrier to Veterans seeking treatment[28] and as an impediment to collecting accurate information from Veterans who are in treatment.[57, 58] This study’s results may partially assuage Veterans’ concerns that openness about substance use will harm PTSD claims.

Several incidental findings in our analysis were of interest. We had anticipated an association between self-reported combat exposure and award outcome because such exposure substantially reduces the amount of corroborating evidence needed to approve a claim.[59] A possible reason we did not find this association (even in a post-hoc analysis in which we added it to the logistic regression) is that we defined combat exposure by Veteran self-report of having been in a combat zone, and 89% of Veterans reported such exposure. However, the exposure that furthers a claim is more specific: PTSD diagnosed in-service, or when the stressor is directly related to engagement in combat with the enemy, experience as a prisoner of war, fear of hostile military activity, or military sexual trauma (MST). In this small, predominantly White sample, we did not replicate previously-reported findings that African American Veterans’ service-connection claims are less likely to be awarded.[60] It is noteworthy that 58% of Veterans reported risky substance use during the preceding 90 days in the confidential research assessments but it was not mentioned in the examiners’ reports. This might reflect it not having been known by the examiner, or having been known but not documented.

It is noteworthy that an important predictor of service-connection award was who the examiner was—clustering within examiner accounted for 16% of the variance in service-connection award outcomes. The effect of clustering within examiner is consistent with other literature about how claims are reviewed. A report from the VA Office of Inspector General found that part of the regional variation in compensation payments was attributable to inconsistent examiner reports (the DBQ template rolled out in 2011 attempted to standardize these reports).[3, 61] There is considerable variability in the way examiners conduct mental health C&P examinations that would be expected to manifest itself in systematic variation in claims decisions between examiners.[2] In a study comparing service-connection status (i.e., connected for PTSD, other mental health, or denied) with an independent semi-structured diagnostic interview, Marx and colleagues found that for a significant minority of Veterans, PTSD diagnostic and service-connection status were discordant.[42] While literature suggests wide variation in preferences, practices, and beliefs among examiners, Speroff and colleagues found that standardized testing in C&P exams greatly increased accuracy and quality of exams, and nearly eliminated variation between examiners.[62]

The study has several limitations. One limitation is that substance use evaluations in C&P exams were not standardized, and whether examiners mentioned risky substance use in reports may reflect examiner factors such as the thoroughness of examiners’ questioning about, documenting of, and interpretations of substance use. But the fact that substance use was mentioned in an examiner’s report does indicate what the examiner knew and described about substance use, and thus is relevant to the question of how the use was associated with the claims decision. Further, when testing assumptions of independence for comparisons of potential covariates between veterans with and without risky substance use mentions, we assessed whether clustering of veterans within clinician accounted for variance in the probability of having clinician-documented risky substance use in the C&P exam report, and the effect of clustering on this variable was not different from zero. The absence of significant clustering on this outcome suggests clinicians did not systematically differ in their recording of substance use on exams. A second limitation is that although claims decisions by VBA are usually based on the information in the examiners’ reports, VBA officials have access to military and medical records. How much other sources of information beyond the examiner’s report were related to the award determination was not considered. Third, we cannot rule out additional, unknown variables that explain the study results. For example, impulsivity might both cause more impairment and be associated with risky substance use. However, whatever the lurking variable might be, we would have to posit that it did not act primarily by impacting the overall CAPS-IV as the effect of risky substance use was present even when controlling for CAPS-IV-measured PTSD severity. Fourth, interrater reliability of CAPS-IV ratings was not ascertained. It is possible that the research assistants who administered the CAPS-IV rated symptoms as related to PTSD that were better explained by other psychiatric conditions. In post-hoc analyses with a subset of n = 205 participants with complete data on BDI and TBI, the effect of risky substance use mentions on exam outcomes remained statistically significant after controlling for BDI scores and TBI history, in addition to all other covariates. Fifth, we did not collect the detailed data from the examiners’ reports that might more clearly establish a pathway between mentions of risky substance use and the claims decision. For example, direct mentions by examiners that substance use appeared to have been caused by PTSD would further buttress our findings and explain how Veterans’ mentions of risky substance use are interpreted. The most important caveat is that these findings reflected a relatively small sample of post-9/11 Veterans at one VA facility, and therefore may not be generalizable to the population of Veterans applying for C&P for PTSD or to Veterans of other eras. Regional variation exists in the rate[63] and degree[61] of PTSD compensation awarded and a multisite study with a larger population would offer a more complete understanding of the impact substance use may have on PTSD claim outcomes.

Notwithstanding these caveats, we conclude that mentions of risky substance use in PTSD C&P exams for Veterans in this sample were associated with greater odds of being service-connected for PTSD. The association between risky substance use mentions and claims awards is consistent with Veterans Benefits Administration rating guidelines and literature suggesting that substance use is temporally related to PTSD. We hope that publicizing this finding will lead to more Veterans who need treatment for addictions getting it, without concern for jeopardizing their chances of being service-connected for PTSD.
